# Persistent Intrinsic Functional Network Connectivity Alterations in Middle-Aged and Older Women With Remitted Depression

**DOI:** 10.3389/fpsyt.2020.00062

**Published:** 2020-02-21

**Authors:** Jennifer N. Vega, Warren D. Taylor, Jason A. Gandelman, Brian D. Boyd, Paul A. Newhouse, Sepideh Shokouhi, Kimberly M. Albert

**Affiliations:** ^1^ Center for Cognitive Medicine, Department of Psychiatry and Behavioral Sciences, Vanderbilt University Medical Center, Nashville, TN, United States; ^2^ Geriatric Research, Education, and Clinical Center, Veterans Affairs Tennessee Valley Health System, Nashville, TN, United States; ^3^ Vanderbilt University School of Medicine, Nashville, TN, United States

**Keywords:** remitted depression, resting state fMRI, women, salience network (SN), executive control network (ECN)

## Abstract

**Background:**

In younger adults, residual alterations in functional neural networks persist during remitted depression. However, there are fewer data for midlife and older adults at risk of recurrence. Such residual network alterations may contribute to vulnerability to recurrence. This study examined intrinsic network functional connectivity in midlife and older women with remitted depression.

**Methods:**

A total of 69 women (24 with a history of depression, 45 with no psychiatric history) over 50 years of age completed 3T fMRI with resting-state acquisition. Participants with remitted depression met DSM-IV-TR criteria for an episode in the last 10 years but not the prior year. Whole-brain seed-to-voxel resting-state functional connectivity analyses examined the default mode network (DMN), executive control network (ECN), and salience network (SN), plus bilateral hippocampal seeds. All analyses were adjusted for age and used cluster-level correction for multiple comparisons with FDR < 0.05 and a height threshold of *p* < 0.001, uncorrected.

**Results:**

Women with a history of depression exhibited decreased functional connectivity between the SN (right insula seed) and ECN regions, specifically the left superior frontal gyrus. They also exhibited increased functional connectivity between the left hippocampus and the left postcentral gyrus. We did not observe any group differences in functional connectivity for DMN or ECN seeds.

**Conclusions:**

Remitted depression in women is associated with connectivity differences between the SN and ECN and between the hippocampus and the postcentral gyrus, a region involved in interoception. Further work is needed to determine whether these findings are related to functional alterations or are predictive of recurrence.

## Introduction

Depression, particularly in later life, is associated with high disability, increased risk for cognitive decline and dementia, elevated suicide risk, and greater all-cause mortality rates (1). While antidepressant medications and psychotherapy can effectively treat depressive episodes in older adults (2–4), individuals who respond to treatment remain at high risk for future episodes (5, 6). Despite this increased risk of depression recurrence, little is known about neurobiological factors that increase vulnerability to depression recurrence in midlife or older adults.

Converging evidence supports intrinsic brain network dysfunction as an underlying neural mechanism that characterizes depressive episodes and may contribute to the pathogenesis of depression (7). Functional connectivity differences in key intrinsic networks that persist during depression remission may represent a neurobiological basis for vulnerability to depression recurrence in older adults (8). Resting-state functional network studies in Major Depressive Disorder (MDD) and remitted MDD (rMDD) generally focus on the default mode network (DMN), executive control network (ECN), and salience network (9, 10). Although data on network dysfunction is limited in older adults with rMDD, evidence supports functional alterations in adult populations with rMDD (11).

The DMN is a group of brain areas that are more active at rest than during goal-oriented or attentionally demanding tasks (12–14). The DMN is hypothesized to mediate task-independent intrinsic thought, with its anterior hub (MPFC) contributing to self-referential processing (consideration of one’s own thoughts and feelings) and emotion regulation of present states and its posterior hub (PCC) being associated with episodic memory retrieval and scene construction (15, 16). Compared with never-depressed adults, in adult MDD, DMN activity is higher when assessing external stimuli (17) and during maladaptive ruminative self-focus (18). While activity in the DMN is typically negatively correlated with activity in ECN regions (19), in MDD, DMN positive functional connectivity with the ECN is increased (20). This increased positive connectivity between the DMN and ECN may contribute to an underlying bias toward allocating cognitive resources towards internal thoughts at the cost of engaging with external stimuli, clinically present as rumination (21). Many of these findings persist during depression remission, with the DMN exhibiting altered connectivity and fewer connections with other networks (22) and reduced deactivation of DMN regions during cognitive tasks (11).

The ECN is engaged during externally directed cognitive tasks and regulates externally driven attention (23), working memory, decision making, emotional regulation, and conflict resolution (23–25). In MDD, activity in ECN regions is lower in response to negative stimuli (26) and during attempts to regulate emotional responses (27). Studies of MDD and late-life depression often observe reduced within-network connectivity of the ECN (9, 10, 28), although this is not universally reported (29). In rMDD, the ECN exhibits reduced within-network connectivity (30) and attenuated activity during “cold” cognitive tasks but greater neural response to emotional stimuli (31, 32).

The SN is activated in response to various salient stimuli (33) and facilitates switching between the DMN and ECN to shift attention from internal states to external stimuli (34). In MDD, SN regions are generally overresponsive to affective challenges, particularly negatively valenced stimuli (35). MDD is characterized by altered connectivity of the amygdala and insula with frontal and anterior cingulate cortex (ACC) regions but also with regions of the ECN and DMN (20, 36, 37).

The purpose of this study was to test for differences in intrinsic network resting-state functional connectivity in midlife and older women with remitted depression (MDDHx) and women without a history of depression (NoHx). We focused exclusively on women, as depression is more common in women and women with previous depressive episodes remain at high risk for recurrence (6, 38). Based on past work examining intrinsic networks in depressed and remitted populations, we hypothesized that remitted depressed women would exhibit alterations in key functional networks including the CCN, DMN, and SN. Additionally, given the substantial past work implicating hippocampal pathology in depression (39, 40) and the potential for aging pathology in an older cohort of women, we tested for differences in hippocampal functional connectivity.

## Methods

### Participants

Sixty-nine postmenopausal women (Past MDD: n=24; No MDD: n = 45) between the ages of 50 and 75 completed 3T MRI with resting-state acquisition. Participants were recruited for a larger study examining the effects of estradiol replacement on stress response in postmenopausal women with and without a history of depression (8). The results reported here include data from all women who completed baseline neuroimaging before initiating estradiol treatment. Participants were recruited through notices in local media and direct mailings that described the study as having a focus on cognition after menopause. Potential participants completed a screening visit before approval for study inclusion. None of the participants were taking ovarian hormones and all had been at least one year without such treatment.

This study was approved by the Vanderbilt University Institutional Review Board. All participants provided written informed consent.

### Screening

Participants were evaluated for cognitive impairment using the Wechsler Abbreviated Scale of Intelligence (WASI), the Mini-Mental State Exam (41) (MMSE; score ≥ 26), the Brief Cognitive Rating Scale (42) (score ≤ 2), and the Mattis Dementia Rating Scale (43) (minimum score 125) to establish a Global Deterioration Scale score (44) (GDS; score ≤ 1) rating the degree of cognitive impairment. Participants were required to have a GDS score of 1–2 and an MMSE score of greater than 25. No participant scored below 123 on the Mattis scale or below 90 on the WASI; participants were of average or above intelligence with no evidence of dementia or mild cognitive impairment.

Participants were screened for current and past depression, mania, and dysthymia using the partial Structured Clinical Interview for DSM-IV-TR (SCID) (45). Participants were excluded for a history of premenstrual dysphoric disorder or any axis I diagnosis (current or past) other than MDD. Criteria for never-depressed participants (NoHx) were: no current or past episodes that met SCID criteria for MDD, dysthymia, or mania, a current score less than 7 on the Beck Depression Inventory (BDI) (46), and less than 15 on the Beck Anxiety Inventory (BAI) (47). Criteria for prior history of MDD (MDDHx) were: at least one episode in the last ten years that met criteria for MDD on the SCID, no MDD episodes in the last year, current BDI score less than 7, and current BAI less than 15. Participants using antidepressant medications were required to have been on a stable regimen and dose for at least 3 months prior to enrollment (n=6).

### Neuropsychiatric Assessments

The Profile of Mood States (POMS) (48) (McNair et al., 1971) assessed mood prior to MRI. The POMS consists of six subscales (Tension/Anxiety, Depression, Anger/Hostility, Fatigue, Confusion, Vigor/Activity) that are used to calculate a total mood disturbance (TMD) score. For Tension/Anxiety, Depression, Anger/Hostility, Fatigue, Confusion, and TMD, higher scores indicate greater mood disturbance. Conversely, for the Vigor/Activity subscale, higher scores indicate greater levels of enthusiasm and optimism.

### Imaging Parameters

Participants were scanned on a research-dedicated 3.0T Philips Achieva whole-body scanner (Philips Medical Systems, Best, the Netherlands) using body coil radiofrequency transmission and an eight-channel head coil. Structural imaging included a Sagittal T1-weighted 3D Turbo Field Echo Sensitivity Encoding (TFE SENSE) sequence (TR = 9.9 ms, TE = 4.6 ms, FA = 8°, FOV = 256 mm^2^, matrix = 256×256, and 1.0 mm slice thickness with no gap for 140 contiguous slices). Resting-state functional MRI was conducted with eyes open (TR = 1500 ms, TE= 35 ms, FA = 90°, 1 FOV = 240 mm^2^, matrix = 80×80, and 5.0 mm slice thickness with no gap, for 24 slices).

### Functional Connectivity Analysis

Resting-state functional MRI images were preprocessed using the CONN toolbox (version 15.g) in SPM12 (49), including realignment of the functional runs and correction for head motion, co-registration of functional and anatomical images for each participant, normalization of the anatomical and functional images to the standard Montreal Neurological Institute template, and spatial smoothing with a Gaussian filter (6 mm at full width at half maximum). Motion artifacts were further detected by applying the Artifact Detection Toolbox as implemented in CONN. We used the medium settings in CONN to apply a displacement threshold of 0.9 mm and a global signal threshold of Z = 5. The outliers detected by ART were applied as nuisance regressors to censor bad volumes in subsequent steps. To effectively mitigate the effects of head motion, denoising in CONN was conducted using the aCompCor method for white matter (five components extracted) and cerebrospinal fluid (five components extracted) signal and realignment parameters (36) with outlier volumes identified by the Artifact Detection Toolbox. We retained all participants with >5 minutes of scan time after excluding outlier volumes. The resulting blood oxygen level-dependent time series were band-pass filtered (0.01 to 0.1 Hz) to further reduce noise and increase sensitivity.

First- and second-level analyses were conducted using standard methods in the Conn Toolbox (34). We selected canonical network regions for each intrinsic functional network to use as key network seed regions of interest: 1) a DMN seed [PCC (50)], 2) bilateral ECN seeds [dlPFC (23)], 3) bilateral SN seeds [anterior insula (23)], and bilateral hippocampal seeds (51) (**Table 1**). First-level subject functional connectivity maps were entered into a seed-to-whole-brain second-level random-effects analysis of the effect of group. Two-sample t-tests were conducted for differences between the subject groups in whole-brain connectivity for each seed. All analyses were adjusted for age and used cluster-level correction for multiple comparisons with FDR < 0.05 and a height threshold of p < 0.001, uncorrected.

**Table 1:** Network seeds.

Network	Region	Coordinates
Executive Control Network (ECN)	Left dorsolateral prefrontal cortex (dlPFC) Right dorsolateral prefrontal cortex (dlPFC)	−42, 34, 20
		44, 36, 20
Default Mode Network (DMN)	Posterior cingulate cortex (PCC)	1, −55, 17
Salience Network (SN)	Left anterior insula (ant INS) Right anterior insula (ant INS)	−32, 26, −14
		38, 22, −10
Hippocampus	Left hippocampus Right hippocampus	-28, -22, -12
		28, -22, -12

### Statistical Analyses

Statistical analyses were performed using IBM SPSS Statistics for Mac, version 25 (IBM Corp., Armonk, N.Y., USA) to evaluate group differences between NoHx and MDDHx groups. Group demographic differences were evaluated using independent samples t-tests. To compare motion between groups, we extracted the outlier counts, mean motion, and mean global signal for each session from the CONN data. We then ran a t-test on each of these measures.

## Results

The study included 69 women (n = 24 with a history of depression (MDDHx) and n = 45 never-depressed (NoHx)) over the age of 50. The were no significant group differences in age or other demographic measures (**Table 2**).

**Table 2:** Demographics and screening.

	NoHx (n=45)	MDDHx (n=24)	*p*
Age	61.13 (6.37)	61.46 (6.32)	0.84
WASI-FSIQ II	114.82 (12.20)	113.86 (12.07)	0.77
MMSE	29.09 (1.04)	29.08 (1.72)	0.98
BDI	3.27 (3.04)	4.12 (3.76)	0.31
BAI	4.04 (4.07)	4.17 (3.24)	0.90

Values reported in the above table are mean (SD). NoHx, No history of depression; MDDHx, History of depression; WAIS-II FSIQ-2, Wechsler Abbreviated Scale of Intelligence-II Full-Scale IQ-2; MMSE, Mini-Mental State Exam; BDI, Beck Depression Inventory; BAI, Beck Anxiety Inventory; POMS, Profile of Mood States.

**Figure 1:**
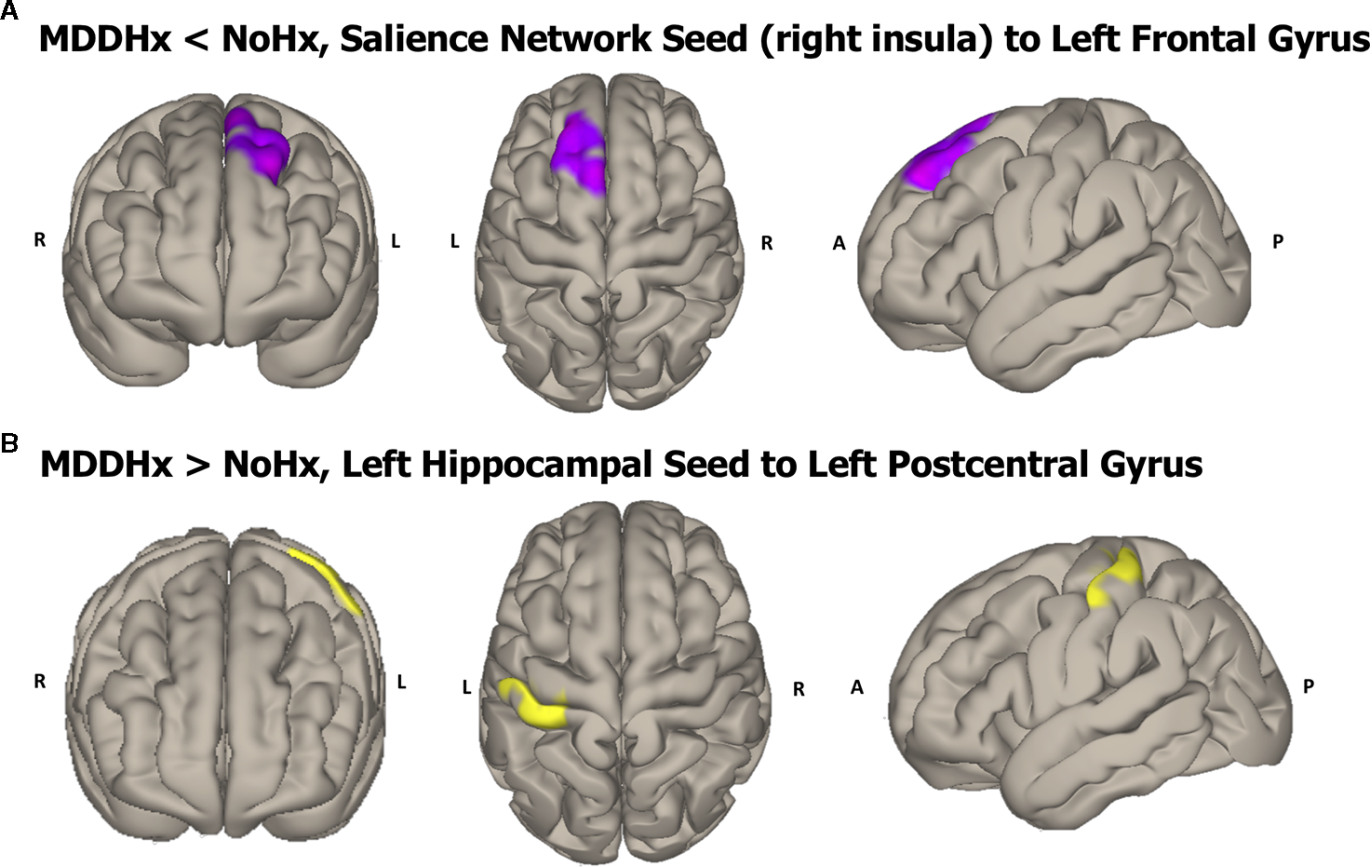
Visualization of the approximate anatomical location of connectivity differences between groups. **(A)** The MDDHx group exhibited decreased functional connectivity between the SN seed (right insula) and the left superior frontal gyrus compared to the NoHx group. **(B)** MDDHx exhibited increased functional connectivity between the left hippocampus seed and the left postcentral gyrus. SN, salience network; NoHx, No history of depression; MDDHx, History of depression; L, left hemisphere; R, right hemisphere; A, anterior; P, posterior.

In whole-brain seed-to-voxel analyses, women with a history of depression (MDDHx) exhibited altered connectivity in two of the examined seeds (**Figure 1**). First, we observed decreased positive functional connectivity between the right insula seed and the left superior frontal gyrus, indicating decreased positive functional connectivity between the SN and ECN hubs. Second, women with a history of depression (MDDHx) exhibited increased positive functional connectivity between the left hippocampus seed and the left postcentral gyrus (see **Table 3**). We did not observe any group differences in functional connectivity for DMN or ECN seeds. To examine the potential effect of antidepressant use, we removed from analyses six participants taking antidepressants at the time of MRI. In this reduced sample (MDDHx n= 19, NoHx n = 44), the results remained unchanged. In addition, there were no differences in head motion parameters between groups (mean motion *t* (67) = -1.09, *p* = 0.28; mean global *t* (67) = -0.27, *p* = 0.79).

**Table 3:** Significant results.

Region Name	MNI Coordinates (x,y,z)	Cluster Size	*p*- uncorrected	*p*-FDR corrected	t-Value
Superior Frontal Gyrus	-10,+24,+46	k =1199	0.000000	0.000000	3.22
Post Central Gyrus	-58, -18, +52	k = 480	0.000008	0.000137	3.22

All analyses were adjusted for age and used cluster-level correction for multiple comparisons with FDR < 0.05 and a height threshold of p < 0.001, uncorrected.

## Discussion

Differences in network functional connectivity are present in women with remitted depression over a year in the past, even while euthymic. These differences appear specific to functional connectivity between the SN and ECN connectivity and the functional connectivity of the hippocampus. We did not observe any differences in functional connectivity for the examined DMN hub regions. Importantly, these differences in network functional connectivity remain when controlling for antidepressant use.

Aberrant resting-state connectivity findings obtained during an acute episode are often conceptualized as a *consequence *of MDD itself, as opposed to representing potential risk factors for depression development or recurrence (52). Past studies in other age ranges report that individuals with rMDD exhibit functional connectivity alterations in the ECN and SN (30, 53) but do not specifically report decreased connectivity between these networks’ hub regions. Studies in adolescents with active MDD found that higher levels of rumination were associated with *reduced* connectivity between the SN and the ECN regions (54). Therefore, the decreased connectivity between the SN and the ECN observed in our rMDD sample could reflect a decreased ability of the ECN to regulate the response to emotional stimuli *via* the SN (8), indicating a vulnerability to future depressive episodes. Alternatively, it could also reflect compensatory changes that promote sustained remission. In addition, the finding of the right anterior insula seed showing differential connectivity could reflect the role the right anterior insula plays in emotional evaluation of stimuli (specifically negative), interoceptive feeling, and visual self-recognition (55).

The observed increase in functional connectivity between the hippocampus and postcentral gyrus and somatosensory cortex may indicate the involvement of interoceptive systems. The hippocampus is involved in aspects of interoception, defined as the perception and interpretation of bodily signals, particularly in the use of interoceptive signals as contextual cues for memory storage and retrieval (56). Previous research shows that interoception plays a critical role in homeostasis, motivated behavior, and emotional processing (55, 57–60). Many models hypothesize that interoceptive processes are altered in depression (61, 62). This observed increase in functional connectivity between these regions could be related to the increased somatization experienced by older adults with depression.

It is important to consider how sex differences influence the neurobiology of remitted and active MDD, including differences in functional connectivity. Most studies that examine sex differences in functional connectivity report greater DMN connectivity in females compared to males in both younger (63–66) and healthy older adults (67). In addition, older males have been shown to have greater SN connectivity than older females (67). These results highlight the importance of considering the effect of sex on resting-state functional connectivity measures, particularly when examining DMN and SN (67). Additionally, the finding of the left hippocampal seed showing differential connectivity could reflect well-documented gender differences in the lateralization of hippocampi between men and women. Studies have demonstrated greater left hippocampal activation in women during memory tasks (68, 69). In addition, a metanalysis demonstrated greater activation of the left hippocampus in studies evaluating emotional processing (regardless of valence) for women compared to men (70), which may reflect enhanced emotional memory in women (71). This is critical for evaluating differences in connectivity for disorders that disproportionally affect females, such as MDD (38). This sex effect may have influenced past reports of altered DMN functional connectivity in rMDD (22, 53), a finding we did not replicate in our sample. The lifetime prevalence of MDD in women is nearly twice that in men (72, 73), with increased vulnerability beginning at puberty and lasting until menopause (73, 74). There are a number of factors that likely contribute to MDD risk, including other neurobiological factors and stressful life events. These factors may either contribute to network alterations or serve as additional risk factors in conjunction with network alterations.

There are several limitations to our analyses. First, this study only included postmenopausal women as participants, which limits the generalizability of these results for young women and men with rMDD. Although sex differences in rMDD have not been extensively reported, prior studies in healthy older adults suggest sex differences in connectivity within DMN and SN (67). This suggests that sex differences could be important to the better understanding of the neurobiological differences inherent in the risk for recurrent MDD. Second, the sample sizes for the diagnostic groups were unequal, which limits the power of our group comparisons and may have reduced our ability to detect group differences. Thus, the results of this study should be interpreted as an exploratory analysis due to the relatively small sample size of the MDDHX group and the unbalanced sample size between the two groups. A third potential limitation of this study is that, because the enrollment criteria for the study required women to be euthymic and not symptomatic, we did not examine any relationships between clinical measures and connectivity, as floor effects would limit our ability to make conclusions. The ranges on clinical measures were small, and there were no significant differences between groups. The relationship between imaging measures and clinical measures is critical. However, for these analyses, we would argue that what is interesting about these findings is that there are differences in functional connectivity between the groups that are not related to clinical symptoms. A fourth limitation of this study is the cross‐sectional examination of remitted depressed individuals, which limits our ability to determine the direction of the study results. Our findings could reflect a decreased ability of the ECN to regulate emotional stimuli *via* the SN; however, they could also reflect compensatory changes that promote sustained remission. A fifth limitation of this study is that a single seed region for the DMN was chosen. Previous studies suggest that the large-scale brain networks such as the DMN can be divided into several subnetworks (75) in neuropsychiatric disorders such as MDD (76). The selection of a single DMN seed could have contributed to the lack of findings in the current study for the DMN. In future, multiple DMN seeds should be evaluated, as alterations in DMN connectivity may exist in other regions.

Further work is needed to determine whether these findings are related to functional alterations and whether they are related to depression recurrence. A longitudinal study examining resting-state functional connectivity differences between individuals with remitted depression and no history of depression will be needed to better determine the directionality of the current study’s findings. Future studies should examine whether the association of depression history and resting-state functional connectivity differs by sex or hormonal status.

## Data Availability Statement

The datasets generated for this study are available on request to the corresponding author.

## Ethics Statement

The studies involving human participants were reviewed and approved by Vanderbilt University Institutional Review Board. The patients/participants provided their written informed consent to participate in this study.

## Author Contributions

PN and KA contributed to the design and implementation of the research. JV, BB, SS, and JG contributed to the analysis of the results.JV and WT drafted the manuscript and designed the figure. All authors discussed the results and commented on the manuscript.

## Funding

Vanderbilt Institute for Clinical and Translational Research (VICTR) CTSA Grant (UL1TR000445) from the National Center for Advancing Translational Sciences to KA, K24 MH110598 and R01 MH102246 to WT, and T32-AG058524 (JV) and the Vanderbilt Memory & Alzheimer’s Center.

## Conflict of Interest

The authors declare that the research was conducted in the absence of any commercial or financial relationships that could be construed as a potential conflict of interest.
